# Machine learning and statistical models for analyzing multilevel patent data

**DOI:** 10.1038/s41598-023-37922-3

**Published:** 2023-08-07

**Authors:** Sunyun Qi, Yu Zhang, Hua Gu, Fei Zhu, Meiying Gao, Hongxiao Liang, Qifeng Zhang, Yanchao Gao

**Affiliations:** 1 Zhejiang Provincial Center for Medical Science Technology and Education Development, Hangzhou, 310000 Zhejiang China; 2 Leuven Statistics Research Centre, Faculty of Science, KU Leuven (Katholieke Universiteit Leuven), 3001 Heverlee, Belgium; 3 Department of Public Utilities Management, Faculty of Humanities and Management, Zhejiang Chinese Medical University, Hangzhou, 310000 Zhejiang China

**Keywords:** Mathematics and computing, Statistics

## Abstract

A recent surge of patent applications among public hospitals in China has aroused significant research interest. A country’s healthcare innovation capacity can be measured by its number of patents. This paper explores the link between the number of patents and ten independent variables. Multicollinearity was carefully detected and removed by using the variable selection method and LASSO regression, respectively. The Poisson model and the negative binomial model were proposed to analyze the patent data. Three goodness of fit tests, the Pearson test, the deviance test, and the DHARMa non-parametric dispersion test, were conducted to investigate if the model has a good fit. After discovering four clusters by conducting agglomerative hierarchical clustering, these two models were replaced by the negative binomial mixed model. The likelihood ratio test was used to determine which model is more appropriate and the results reveal that the negative binomial mixed model outperforms both the Poisson model and the negative binomial model. Three variables, number of health technicians per 10,000 people, financial expenditure on science and technology as well as number of patent applications per 10,000 health personnel, have a significantly positive relationship with the number of patents in Chinese tertiary public hospitals.

## Introduction

Over the past ten years, the number of patents application in China has been skyrocketing. China has surpassed all other countries worldwide in terms of patent application filings since 2011^1^. The number of applications for invention patents climbed from 526,412 in 2011 to 1,586,000 in 2021, with an average annual growth rate of 11.7%, according to the National Bureau of Statistics of China. Healthcare patents, one of the most important areas of the patent, provide important protections for intellectual property in the medical arena, which can further innovations that benefit everyone. Medical patents are defined broadly to include patents that relate to pharmaceuticals; methods of making and using them; medical treatment regimens; surgical procedures; medical devices; health care information technology for hospital and health care management systems; and combinations of them^2^.

Medical patents holders are mainly tertiary public hospitals in China. In China, hospitals are organized in a three-tiered hierarchy, with primary hospitals providing general healthcare and preventive care to the population. Secondary hospitals provide complete health care to a region, accept referrals from primary hospitals, and are also responsible for teaching and research. Lastly, tertiary hospitals, often located in urban areas, are responsible for specialty care and act as medical centres for several regions^3–6^. This system is motivated by the expectation that tertiary hospitals can focus more on research and lead Chinese medical innovation to a higher level. As for the evaluation, patents are vital to measure hospitals’ achievement. The number of applied patents can be related to many factors. For example, based on the scale of a region, the investment or the population size can be considered.

Following the acquisition of data on the number of patents, a linear regression model will be considered first to fit. However, we would only consider the linear regression process, the least absolute shrinkage and selection operator (LASSO) regression model, in the step of variable selection^7^. The number of patents is a counting value over a fixed period of time. In other words, we are counting the occurrences of the event that a patent application is submitted during a certain time interval. We are assuming that the event happens completely randomly and independently. Thus, the number of patents no longer follows a normal distribution, and hence a simple linear regression model will be dismissed under our assumption.

To overcome the problem of the non-normal distribution of dependent variables, generalized linear models like the Poisson regression model will be introduced^8^. A count variable is a variable that reflects the number of occurrences of an interested event in a fixed period of time^8^. Linear regression model is not appropriate for a count variable as a dependent variable and problems like biased standard errors will occur^9^. Poisson regression model offers an alternative analysis for count data. It can also be used for summarizing relative risk across strata of a covariate and for evaluating interactions between covariates^10^. The Poisson regression model is an example of generalized linear models (GLM). There are three components in a GLM: a random component, a systematic component and a link function. Random component is the probability distribution of the dependent variable, for example, Poisson distribution for response variable in the Poisson regression. Systematic component refers to the independent variables as a combination of linear predictors and link function specifies the link between random and systematic components^11–13^. Poisson regression with overdispersion can be replaced with the negative binomial model if the assumption must be met for the model to be valid^14^. Besides, this paper also proposed a mixed model, which is a combination of regression with clustering^15–17^. The summary of results is provided in the last part.

## Materials and methods

### Data description

Patent number data from the effective patents for tertiary public hospitals in China was used as the dependent variable in this paper. The Baiten database (www.baiten.cn) was thoroughly searched for patent numbers in tertiary public hospitals. Since a time lag exists between the application time and publication time in patent authorization period^18^, we select the year of application to conduct our count procedure. A total number of 165,262 patent was collected in 2243 tertiary public hospitals from year 2016 to year 2021. The independent variables, population, GDP, number of health technicians per 10,000 people, R &D, number of health personnel, financial expenditure on education, financial expenditure on science and technology, financial health care expenditure, health industry income per capita and number of patent applications per 10,000 health personnel, were obtained from the National Bureau of Statistics (NBS) website.

**Figure 1:**
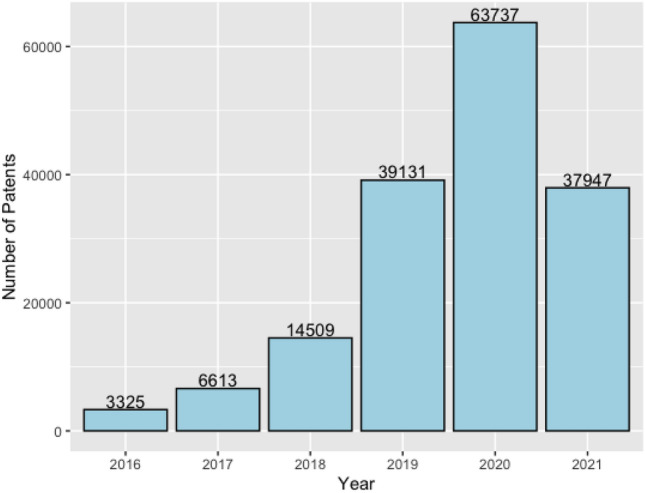
Bar plot of the number of patents every year between 2016 and 2021.

Summary statistics regarding variables in our data are shown in Table 1. Table 2 and Fig. 1show the number of patents from the year 2016 to the year 2021. The sharp increase in the number of patents in 2018 was due to the regulation announcement that tertiary public hospitals began assessing patents in 2018. The reason why only a half number of patents in 2021 compared with the year 2020 is that China carried out intellectual property quality improvement projects.

**Table 1:** Variable description.

Abbreviations	Description	Mean	SD	Min.	Max.
NumPat	Number of patents	888.51	1446.50	0.00	9,951.00
Pop	Population	4526.72	2975.69	340.00	12,684.00
GDP	Gross domestic product	30,253.57	24,993.020	124,369.70	1173.00
NumHeaTec	Number of health technicians per 10,000 people	71.05	12.98	45.00	126.00
R &D	Research and development	99,422.66	144,492.69	190.00	700,017.00
NumHeaPer	Number of health personnel	40.37	25.34	2.92	102.79
FinEdu	Financial expenditure on education	1000.06	616.57	152.57	3,510.56
FinSci	Financial expenditure on science and technology	166.32	201.56	4.81	1168.79
FinHea	Financial health care expenditure	510.84	300.43	69.97	1772.99
HeaInc	Health industry income per capita	99,681.45	31,336.74	51,135.00	208,481.00
NumPatHeaPer	Number of patent applications per 10,000 health personnel	17.99	22.05	0.00	120.87
Pro	Province	categorical	categorical	categorical	categorical

**Table 2:** The number of patents from 2016 to 2021.

Year	Number of patents
2016	3325
2017	6613
2018	14,509
2019	39,131
2020	63,737
2021	37,947

### Variable selection

Multicollinearity appears when independent variables (NumPat, Pop, GDP, NumHeaTec, R &D, NumHeaPer, FinEdu, FinSci, FinHea, HeaInc and NumPatHeaPer) in the regression model are highly correlated to each other. It generates high variance of the estimated coefficients and hence, the coefficient estimates corresponding to those interrelated explanatory variables will lead to wrong results. To detect multicollinearity, the correlation matrix is plotted. Variable selection and LASSO regression can be utilized to eliminate the problem of multicollinearity.

#### Variable selection via variance inflation factor

One of the two methods we applied here is variable selection method using variance inflation factor (VIF).

VIF is used to measure the multicollinearity among the our ten independent variables. When there is high correlation among the predictor variables, the standard errors of predictors coefficients will increase, then the variance of their coefficients will be inflated.

The VIF on NumPat, Pop, GDP, NumHeaTec, R &D, NumHeaPer, FinEdu, FinSci, FinHea, HeaInc, NumPatHeaPer is defined as

1 $$\begin{aligned} VIF_j =\frac{1}{1-R_j^2} \end{aligned}$$

where $R_j$ is the coefficient determination for the regression of $x_j$ on the remaining variables^19^. For example, to calculate $R_{NumPat}^2$, we regress *NumPat* against all other independent variables and then we can obtain $R_{NumPat}^2$, thus, VIF of *NumPat* can be derived. Normally, for VIF, a value of 10 and above indicates multicollinearity^20^.

The variable selection procedure is done by removing the variable that has the highest VIF value. Then correlation matrix and VIF were calculated again to remove next variable that has the highest VIF value. The procedure was stopped when there is no VIF value larger than 10^21^.

#### LASSO regression

Though variable selection via VIF can perform feature selection and make parsimonious models, with advancements in machine learning, Least Absolute Shrinkage and Selection Operator, known as LASSO, provides a good alternative as it gives much better output, requiring fewer tuning parameters and being automated to a large extend^22^.

LASSO was proposed by Tibshirani (1996) for subset selection based in regression process. It puts a constraint on the sum of the absolute values of the model parameters. In other words, it apply a shrinking or regularization process where it penalizes the coefficients of the regression variables, shrinking some of them to zero.

LASSO solves the $l_1$-penalized regression problem of finding $\beta ={\beta _j}$ to minimize

2 $$\begin{aligned} \sum _{i=1}^N\left( y_i-\sum _j x_{i j} \beta _j\right) ^2 +\lambda \sum _{j=1}^p\left| \beta _j\right| \end{aligned}$$

where $x_{i j}$ are the standardized NumPat, Pop, GDP, NumHeaTec, R &D, NumHeaPer, FinEdu, FinSci, FinHea, HeaInc, NumPatHeaPer and $y_{i}$ is the NumPat. The method of K-fold cross-validation is performed to tuning parameter values and we used ten-fold. This procedure begins with splitting the data into “folds.” Then the prediction performance of each model is evaluated across the “left-out” fold using a sequence of tuning parameter settings. This procedure is done until every fold has been calculated as test data. Typically, the tuning parameter is set to the sequence value with the smallest cross-validation error^23^.

### Methods

The Poisson regression model was utilized to analyze the relationship between count data and the independent variables. In Poisson regression, the response variable is assumed to follow a Poisson distribution ${NumPat} \sim Poisson(\lambda _i)$, and hence the regression formula is defined as

3 $$\begin{aligned} \log (\lambda _i)=\beta _0+\varvec{\beta ^{\prime }} {\textbf{x}}=\beta _0+\beta _1 x_{i1}+...+\beta _p x_{ip}=\beta _0+\sum _{j=1}^p\beta _j x_{ij} \end{aligned}$$

or written as

4 $$\begin{aligned} \lambda _i=E(y_i \mid {\textbf{x}})=e^{\beta _0+\varvec{\beta ^{\prime }} {\textbf{x}}}=e^{\beta _0+\beta _1 x_{i1}+...+\beta _p x_{ip}}=e^{\beta _0+\sum _{j=1}^p\beta _j x_{ij}} \end{aligned}$$

where $\lambda _i$ is the expected value or mean of the NumPat, ${\textbf{x}}$ is a matrix of the NumPat, Pop, GDP, NumHeaTec, R &D, NumHeaPer, FinEdu, FinSci, FinHea, HeaInc, NumPatHeaPer and $\beta _0$, and $\varvec{\beta ^{\prime }}$ is the set of regression coefficients to be estimated.

Different from linear regression models, the Poisson regression model uses Maximum Likelihood Estimation (MLE) method instead of OLS estimation. Since we have assumed that ${NumPat} \sim Poisson(\lambda _i)$, the probability density function (pdf) of $y_i$ (NumPat) is

5 $$\begin{aligned} f(y_i \mid {\varvec{x}};\lambda _i)=\frac{e^{-\lambda _i}\lambda _i^{y_i}}{y_i!}, y_i=0,1,2,... \end{aligned}$$

then the likelihood function takes the form

6 $$\begin{aligned} L(\varvec{\beta } \mid {\varvec{x}}, {\varvec{y}})=\prod _{i=1}^N\frac{e^{y_i {\varvec{x}}_i^T \varvec{\beta }} e^{-e^{{\varvec{x}}_i^T \varvec{\beta }}}}{y_i!} \end{aligned}$$

given N vectors ${\varvec{x}}_i \in {\mathbb {R}}^{p+1}$ with $i=1,...,N$ along with a set of N values $y_1,...,y_N \in {\mathbb {N}}$. Hence the log-likelihood is derived as

7 $$\begin{aligned} \ell (\varvec{\beta } \mid {\varvec{x}}, {\varvec{y}})= \sum _{i=1}^N (y_i {\varvec{x}}_i^T \varvec{\beta } - e^{{\varvec{x}}_i^T \varvec{\beta }} - log(y_i!)) \sim \sum _{i=1}^N (y_i {\varvec{x}}_i^T \varvec{\beta } - e^{{\varvec{x}}_i^T \varvec{\beta }}) \end{aligned}$$

and finally some calculation methods would be taken to maximize the log-likelihood, selecting the best values of $\varvec{\beta }$.

The interpretation of $\varvec{\beta ^{\prime }}$ is that, in particular, given one unit change of the independent variable, the difference in the natural logarithm of expected counts is expected to change by the respective regression coefficient, with other independent variables in the model constant.

We should recognize that models can only approximate complete information or reality. Thus, we tried to find the model that minimize the loss of information. A useful criterion in model selection named Akaike’s information criterion (AIC) is defined as

8 $$\begin{aligned} \textrm{AIC}=-2[\mathrm {~L}(\beta )]+2 \textrm{k} \end{aligned}$$

where $\mathrm {~L}(\varvec{\beta })$ is the log-likelihood function of the candidate model evaluated under $\varvec{\beta }$ by using observations and *k* is the number of unknown parameters. Then, the resulting model is subjected to the log-likelihood value where $\mathrm {~L}(\varvec{\beta })$ is log-likelihood at convergence.

A basic assumption of the Poisson distribution is that, for the count data, the mean equals the variance. However, this assumption is not always satisfied. Real data often does not show this specific pattern and the variance tends to be larger than the mean for most of the data. This is known as overdispersion problem in statistics^24^.

We have discussed that it is unrealistic to assume $Var(y_i)=E(y_i)$ for most cases, hence a more flexible relationship between the variance and the mean should be considered^25^. As a result, the negative binomial model is proposed.

In the negative binomial model, the number of patents $y_i$ was assumed to follow the negative binomial distribution, that is, $y_i \sim N B\left( y_i \mid \lambda _i, \theta \right)$ with $\lambda _i \sim Gamma(\theta ,\theta e^{-{\varvec{x}}_i^T \varvec{\beta }})$, whose pdf is

9 $$\begin{aligned} f(y_i \mid \theta )=\frac{\Gamma \left( y_i+\theta \right) }{\Gamma (\theta ) y_{i} !} \cdot \left( \frac{\theta }{e^{{\varvec{x}}_i^T \varvec{\beta }} +\theta }\right) ^\theta \cdot \left( \frac{e^{{\varvec{x}}_i^T \varvec{\beta }} }{e^{{\varvec{x}}_i^T \varvec{\beta }}+\theta }\right) ^{y_i} \end{aligned}$$

where $\lambda _i$ is the mean and $\theta$ is the dispersion parameter that controls the amount of overdispersion. Thus, the variance and the mean of $y_i$can be derived as

10 $$\begin{aligned} E(y_i)&=\mu =E[E(y_i \mid \lambda _i)]=E(\lambda _i)=e^{{\varvec{x}}_i^T \varvec{\beta }} \end{aligned}$$

11 $$\begin{aligned} Var(y_i)&=E[Var(y_i \mid \lambda _i)]+Var(E[y_i \mid \lambda _i])=E(\lambda _i)+Var(\lambda _i)=e^{{\varvec{x}}_i^T \varvec{\beta }}+\frac{1}{\theta } e^{2{\varvec{x}}_i^T \varvec{\beta }}=\mu + \frac{1}{\theta }\mu ^2 \end{aligned}$$

As the above formula shows, the variance and the mean are not the same thing in the negative binomial regression assumption. This is not the case with the Poisson regression. Instead, the model assumes a quadratic relationship between the mean and the variance. The negative binomial model can be used for overdispersed count data and it can be considered as a generalization of Poisson regression since it has the same mean structure as Poisson regression and it has an extra parameter to model the overdispersion.

The MLE of parameters in the negative binomial model is straightforward^25^. Lawless has discussed about the efficiency and robustness properties of the inference procedure^26^.

Hierarchical clustering, one of the most popular unsupervised learning methods, was conducted to find if there exists correlation in the data. In other words, within-cluster observations have high similarity in comparison to others. There are two approaches, agglomerative hierarchical clustering and divisive hierarchical clustering. Agglomerative hierarchical clustering is a bottom up approach that each observation starts from its own cluster, and then clusters are grouped as observations move up the hierarchy. Divisive hierarchical cluserting is a top down approach that observations start from one cluster, and then split as observations moving down the hierarchy. However, the complexity of divisive clustering is ${\varvec{O}}(2^n)$, which makes it too slow for large data sets. Moreover, no provision can be made for a relocation of observations that may have been ’incorrectly’ grouped at an early stage in divisive hierarchical cluserting^27^. Therefore, the agglomerative clustering was used to group data into clusters based on their similarity in this paper.

Euclidean distance matrix was used for hierarchical clustering and the linkage criterion determines the distance between sets of observations as a function of the pairwise distances between observations. Ward’s method is based on the objective of minimizing the deterioration in the overall within sum of squares. The latter is the sum of squared deviations between the data in a cluster and the centroid:

12 $$\begin{aligned} WSS=\sum _{i \in C}\left( x_i-{\bar{x}}_C\right) ^2 \end{aligned}$$

with ${\bar{x}}_i$ is a data point and${\bar{x}}_C$ as the centroid of cluster *C*. Since any merger of two existing clusters results in an overall WSS decline, Ward’s method is intended to minimize this decline. In other words, it is utilized to minimize the difference between the new WSS in the merged cluster and the total WSS of the merged components.

Dendrogram is widely used in visualizing a clustering hierarchy and it is simple to interpret similarity and clustering. The horizontal axis of the dendrogram indicates the dissimilarity or distance between clusters, while the vertical axis represents the objects and clusters.

The Poisson mixed effects model can be an appropriate choice for clustered patent count data. However, it still suffers from the overdispersion problem. Therefore, the negative binomial mixed model was proposed based on the negative binomial model, to analyze the clustered count data, where the observations are no longer considered as independent with each other but are correlated on the counts. Observations are divided into several clusters. These clusters are also called “subjects” and observations in each cluster are seen as repeated measurements over a period of time, hence they have correlation, while observations from different clusters or subjects are independent. To be more specific, $y_{ij}$ is the value of the patent count variable for $i_{th}$ subject at $j_{th}$ time point.

Compared with the negative binomial model, the only change in the negative binomial mixed model is that, in order to take the influence of within-cluster correlation into account, we add subject-specific random effects into the linear predictor. The mean of $i_{th}$ subject $\mu _i$ is related to the host variables via the logarithm link function written in matrix notation:

13 $$\begin{aligned} \log \left( \mu _i\right) =X_i \varvec{\beta }+Z_i {\varvec{b}}+ \epsilon _i, {\varvec{b}} \sim {\varvec{N}}(0, \varvec{\Psi }) \end{aligned}$$

where $\varvec{\beta }$ is the vector of fixed effects for the independent variables (NumPat, Pop, GDP, NumHeaTec, R &D, NumHeaPer, FinEdu, FinSci, FinHea, HeaInc, NumPatHeaPer)$X_i$, cluster${\varvec{b}}$ is the vector of random effects and $\epsilon _i$ is the random errors. The random effects are utilized to partition the multiple sources of variation, and thus to avoid biased inference on the effects of the NumPat, Pop, GDP, NumHeaTec, R &D, NumHeaPer, FinEdu, FinSci, FinHea, HeaInc and NumPatHeaPer. The vector of the random effects is usually assumed to follow the multivariate normal distribution and $\varvec{\Psi }$ is a positive-definite variance-covariance matrix that determines the form and complexity of random effects b.

## Results

### Variable selection

**Figure 2:**
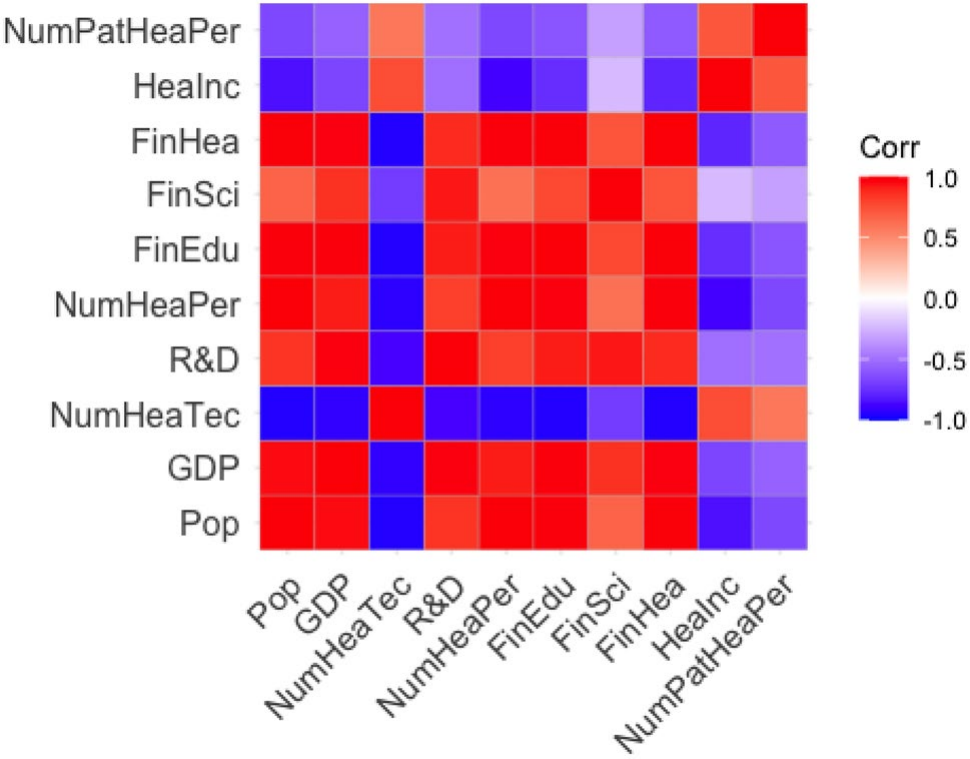
Correlation matrix heatmap for measuring of dispersion.

The correlation matrix plot was plotted to detect if multicollinearity exists. According to the heat map in Fig. 2, obviously there is severe collinearity among the predictor variables. Thus, before fitting models, some measures may need to be taken to avoid this problem, otherwise we might have irrelevant variables in our model, influencing the significance of coefficients.

A more formal way to test multicollinearity is the Variance Inflation Factor. The multicollinearity arises when VIF is larger than 10 and the detailed VIF results are shown in Table 3. A variable selection procedure was conducted to get rid of the high multicollinearity problem. It is done by removing the variable that has the highest VIF value, which is variable Population. Then the correlation matrix and VIF were calculated again to remove the next variable that has the highest VIF value. The procedure was stopped when there was no VIF value larger than 10. At the end of our procedure, the number of health technicians per 10,000 people, R &D, number of health personnel, financial expenditure on science and technology, health industry income per capita and number of patents applications per 10,000 people can be used with a further regression model, and the VIF values of these variables are shown in Table 4.

**Table 3:** VIF with all variables.

Variable	VIF	Detection
Pop	107.34	Collinearity
GDP	28.96	Collinearity
NumHeaTec	4.33	No collinearity
R &D	13.58	Collinearity
NumHeaPer	94.16	Collinearity
FinEdu	43.29	Collinearity
FinSci	12.85	Collinearity
FinHea	32.37	Collinearity
HeaInc	3.76	No collinearity
NumPatHeaPer	2.63	No collinearity

**Table 4:** VIF with selected variables.

Variable	VIF
NumHeaTec	1.78
R &D	5.58
NumHeaPer	2.55
FinSci	5.98
HeaInc	3.40
NumPatHeaPer	1.98

In LASSO regression, 5-fold cross-validation is performed to find an optimal lambda value, which equals 0.01. After using this optimal lambda value for LASSO regression, we have the coefficients of population, GDP, financial expenditure on education and Financial health care expenditure constrained to 0, leaving number of health technicians per 10,000 people, R &D, number of health personnel, financial expenditure on science and technology, health industry income per capita and number of patents applications per 10,000 health personnel. It is the same result when we use the variable selection method via the VIF.

### Poisson regression model and negative binomial model

After backward selection based on the AIC used, the number of health personnel was dropped and we used left variables to generate our Poisson regression model, negative binomial model and negative binomial mixed model. Goodness of fit tests are performed to check if the model is correct given the data. First, the Pearson and deviance goodness of fit tests were utilized to test the goodness of fit of the Poisson regression model. The p-values of both these two tests are close to 0. Thus, we don’t have enough evidence to show our model is good. Moreover, Kolmogorov-Smirnov test were used to test the goodness of fitness. The p-value of KS test is close to 0 and the residuals of the Poisson model didn’t follow a uniform distribution.

DHARMa non-parametric dispersion has a very nice test for dispersion and the result showed that the observed value was much larger than what we could expect under the model. Thus, the Poisson regression model suffers from overdispersion.

**Figure 3:**
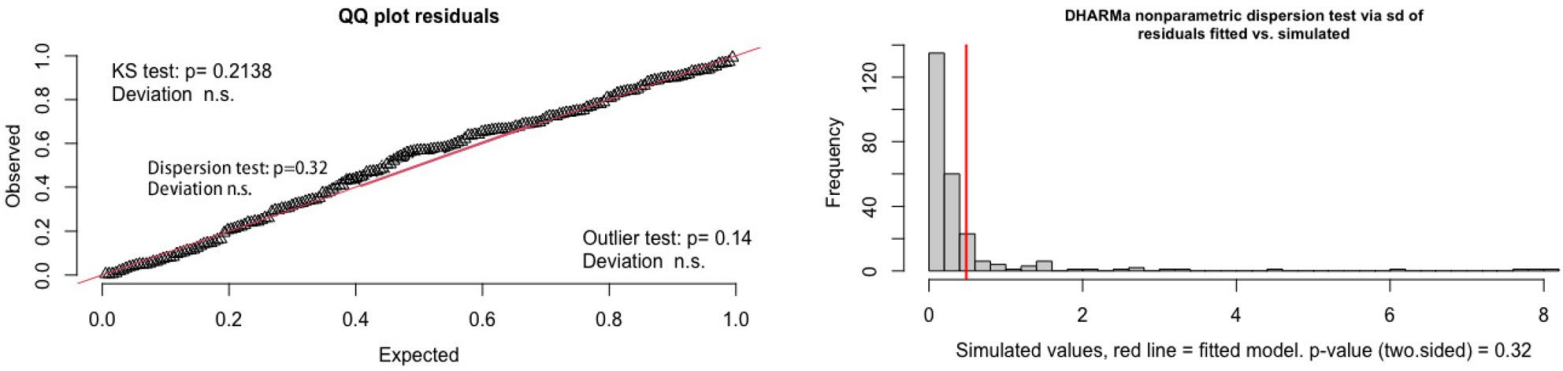
Goodness of fit for negative binomial model. Left: the Kolmogorov-Smirnov test indicates that the residuals of the negative binomial model follow a uniform distribution; Right:the DHARMa nonparametric dispersion test shows that the overdispersion problem no longer exists in the negative binomial model.

In order to deal with the overdispersion problem, a negative binomial model was used. As we can see from the Fig. 3, the *p*-value of the Kolmogorov-Smirnov test showed the residuals of the negative binomial model follow the uniform distribution and the DHARMa nonparametric dispersion test showed the overdispersion problem no longer exists.

### Hierarchical clustering

**Figure 4:**
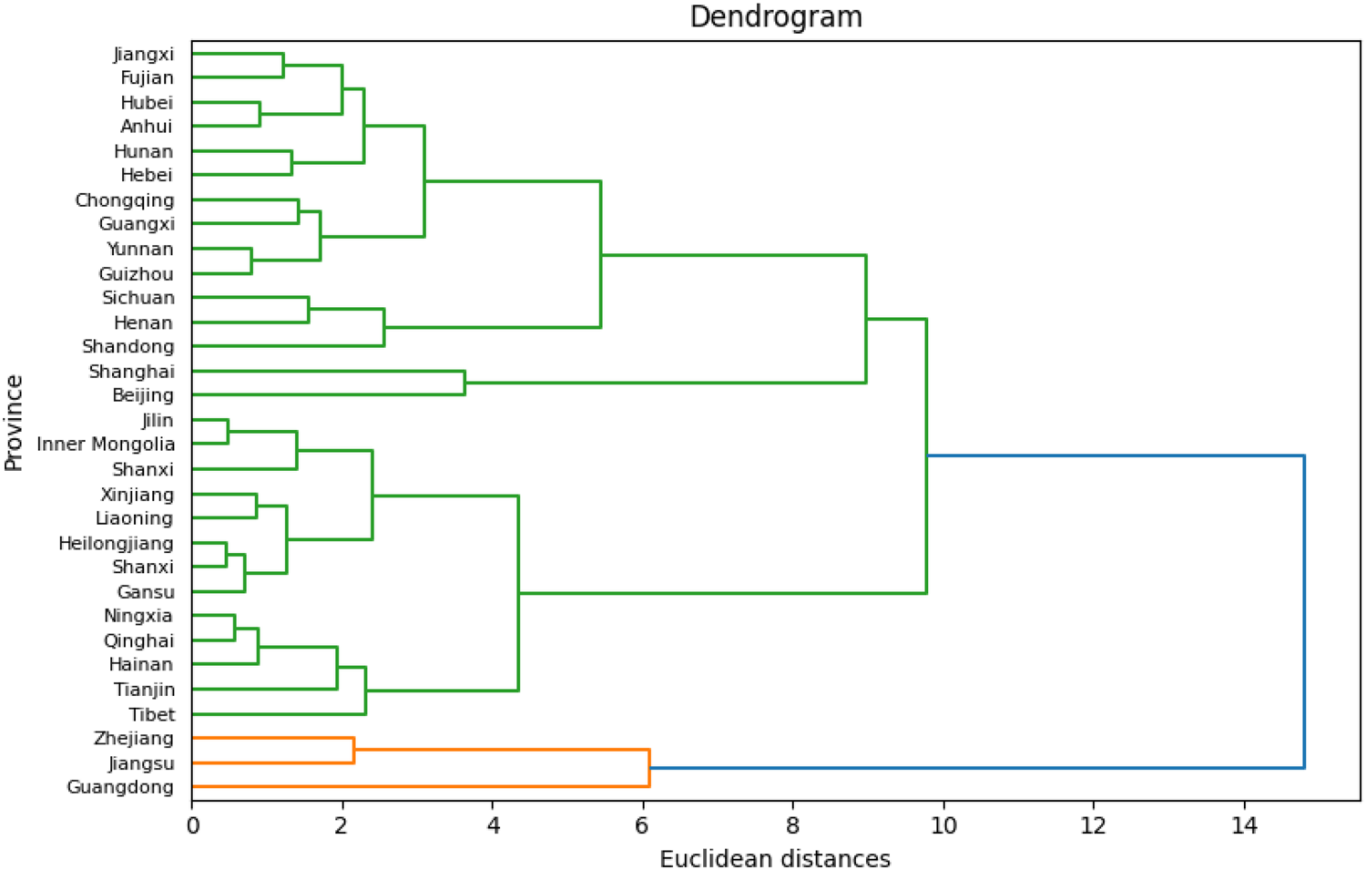
Dendrogram of a hierarchical clustering using agglomerative clustering.

To detect if there are correlations or clusters among the 31 provinces in China, the data of the year 2021 was selected to conduct the hierarchical clustering method. After plotting the dendrogram in Fig. 4, four clusters can be summarized from the dendrogram plot. After doing agglomerative hierarchical clustering among the 31 provinces, and the detailed provinces in the four clusters are shown in Table 5.

**Table 5:** Clustering results.

Cluster	Province
Cluster 1	Jiangsu, Zhejiang, Guangdong
Cluster 2	Tianjin, Shanxi, Neimenggu, Liaoning, Jilin, Heilongjiang, Hainan, Xizang, Shanxi, Gansu, Qinghai, Ningxia, Xinjiang
Cluster 3	Beijing, Shanghai
Cluster 4	Hebei, Anhui, Fujian, Jiangxi, Shandong, Henan, Hubei, Hunan, Guangxi, Chongqing, Sichuan, Guizhou, Yunnan

### Negative binomial mixed model

Taking the clustering and the overdispersion problem into account, a negative binomial mixed model was conducted in this paper. The results of the coefficient estimator and *p*-values of Poisson model, negative binomial model (abbreviated as NBM) and negative binomial mixed model’s fix effect (abbreviated as NBMM FE) are shown in Table 6.

**Table 6:** Model Estimated Cficients.

Variable	Poisson		NBM		NBMM FE	
	ES (95% CI)	*p*-value	ES (95% CI)	*p*-value	ES (95% CI)	*p*-value
NumHeaTec	0.20 (0.20,0.21)	< 0.001	0.35 (0.16,0.55)	< 0.001	0.72 (0.54,0.92)	< 0.001
R &D	0.06 (0.05,0.06)	< 0.001	0.39 (0.06,0.72)	< 0.01	0.34 (− 0.08,0.76)	0.11
FinSci	0.34 (0.33,0.35)	< 0.001	0.20 (− 0.14,0.56)	0.2	0.36 (0.03,0.70)	< 0.05
HeaInc	− 0.32 (− 0.33, − 0.31)	< 0.001	− 0.41 (− 0.64,-0.17)	< 0.001	− 0.38 (− 0.57,-0.18)	< 0.001
NumPatHeaPer	0.64 (0.64,0.65)	< 0.001	1.31 (1.09,1.55)	< 0.001	1.12 (0.92,1.32)	< 0.001

A likelihood ratio test was used to determine if the negative binomial model is more appropriate statistically than the Poisson model, and the results <0.001 suggest that the negative binomial model is a better fit. The same conclusion can be drawn from the Pearson goodness of fit test (*p*-value = 0.98), deviance goodness of fit test (*p*-value = 0.05) and the DHARMa non-parametric dispersion test (*p*-value = 0.32) in the negative binomial model. The likelihood ratio test between the negative binomial model and the negative binomial mixed model suggests that the multilevel model provides a better fit. The variance of the random effects equals 0.2908, which is not too close to 0 and thus, we cannot assume all provinces are independent of each other and the clustering needs to be considered in this data.

The AIC of three models, Poisson model, negative binomial model and negative binomial mixed model, are 71270, 2588.1 and 2533.6, respectively. It is also proved that negative binomial mixed model outperformed than these two models in our data.

## Discussion

Regarding to the count variable, number of patents, Poisson regression model is a commonly used analysis. The Poisson regression is performed based on the assumption that the mean and the variance of the dependent variable are the same. Overdispersion appears when there is more variability around the variance than the mean^28^. Therefore, the negative binomial model is better than the Poisson model when the data shows evidence of overdisperson^29,30^. After conducting the agglomerative hierarchical clustering, four clusters among the 31 provinces were demonstrated in Table 5 and Fig. 4. Therefore, we have solid evidence to take the clustering into account and use multilevel model in this data. The R &Ds of Jiangsu, Zhejiang and Guangdong provinces are almost 10 times larger than other provinces which is the primary reason that these three provinces are grouped. It means that Jiangsu, Zhejiang and Guangdong have a relatively high economy and undertake to innovate and introduce new products and services. Beijing and Shanghai are the two major and most well-known provinces in China. They have the similar economic status, policies and wealth structures. According to our data, all of variables are similar between Beijing and Shanghai and it is reasonable to group them together. Another two clusters can be summarized by saying that they are separated by region—most of the provinces in cluster 2 are from northern China and most of the provinces in cluster 4 are from southern China.

Considering both the clustering and the overdispersion problem, a negative binomial mixed model was proposed. The comparison among estimated coefficients with confidence intervals and p-values of the Poisson model, negative binomial model and negative binomial mixed model’s fix effect are shown in Table 6. From the results of negative binomial model, three variables, number of health technicians per 10,000 people, financial expenditure on science and technology as well as number of patents applications per 10,000 health personnel, have significantly positive relationship with the number of patents in Chinese tertiary public hospitals. To be specific, holding all other variables constant in the negative binomial mixed model , by increasing the number of patents applications per 10,000 health personnel by 1 unit, the number of patents will increase more than two times . The variable, health industry income per capita, has a significantly negative relationship with the patent number and when it increases by 1 unit, the number of patents decreases by 32%. Moreover, the likelihood ratio test and AIC are used to compare two models based on the ratio of the likelihoods^31,32^. The results of likelihood ratio test revealed that negative binomial mixed model outperformed both the Poisson model and the negative binomial model in the data. The variance of the random effects in the negative binomial model is not close to 0 and it also proved that there were associations among the provinces.

The reason why R &D is not significant in the negative binomial mixed model is that we add the province as a random effect in this model. This result is consistent with the finding that R &D is very imprecisely estimated when comparing France and the USA^33^. It may be because the R &D is a significant variable when we group the provinces, but after we clustered, each group had close R &D and thus, the p-value of R &D is not significant after we grouped.

We suggest that each province should adjust the proportion of financial investment and health technicians in different regions according to the local conditions. For example, relatively wealthy provinces can devote more resources to increase the number of health technicians and the protection and supervision of the patent exchange market, while less wealthy provinces should invest more finance in medical science and technology to increase their research and development ability.

The literature on comparison in terms of the traditional variable selection method and the LASSO regression remains scarce. In our study, we compared these methods in the selection of the feature. Moreover, to the best of our knowledge, this paper is the first work to use hierarchical clustering before conducting the multilevel model. One potential limitation of our negative binomial mixed model is that it is not designed to explicitly detect the association between the number of patents and one specific independent variable. The variance function versus the observed variance in our negative binomial mixed model needs to be further investigated and if the variance is wrong, the random effects need to be fixed since it models the variance and covariance structure.

## Data Availability

The datasets generated during and analysed during the current study are available from the corresponding author on reasonable request.
